# Pogostone Enhances the Antibacterial Activity of Colistin against MCR-1-Positive Bacteria by Inhibiting the Biological Function of MCR-1

**DOI:** 10.3390/molecules27092819

**Published:** 2022-04-28

**Authors:** Shengnan Xie, Li Li, Baihe Zhan, Xue Shen, Xuming Deng, Wenxi Tan, Tianqi Fang

**Affiliations:** 1 Key Laboratory of Zoonosis Research, Ministry of Education, Institute of Zoonosis, College of Veterinary Medicine, Jilin University, Changchun 130062, China; tigerxsn@jlu.edu.cn (S.X.); lilitonghappy@163.com (L.L.); zhanbh9920@jlu.edu.cn (B.Z.); dengxm@jlu.edu.cn (X.D.); 2 Department of Food Quality and Safety, Jilin University, Changchun 130062, China; shenxue417@163.com; 3 Department of Obstetrics and Gynecology, The Second Hospital of Jilin University, Changchun 130041, China

**Keywords:** MCR-1, pogostone, colistin, *Enterobacteriaceae*

## Abstract

The emergence of the plasmid-mediated colistin resistance gene *mcr-1* has resulted in the loss of available treatments for certain severe infections. Here we identified a potential inhibitor of MCR-1 for the treatment of infections caused by MCR-1-positive drug-resistant bacteria, especially MCR-1-positive carbapenem-resistant *Enterobacteriaceae* (CRE). A checkerboard minimum inhibitory concentration (MIC) test, a killing curve test, a growth curve test, bacterial live/dead assays, scanning electron microscope (SEM) analysis, cytotoxicity tests, molecular dynamics simulation analysis, and animal studies were used to confirm the in vivo/in vitro synergistic effects of pogostone and colistin. The results showed that pogostone could restore the bactericidal activity of colistin against all tested MCR-1-positive bacterial strains or MCR-1 mutant–positive bacterial strains (FIC < 0.5). Pogostone does not inhibit the expression of MCR-1. Rather, it inhibits the binding of MCR-1 to substrates by binding to amino acids in the active region of MCR-1, thus inhibiting the biological activity of MCR-1 and its mutants (such as MCR-3). An in vivo mouse systemic infection model, pogostone in combination with colistin resulted in 80.0% (the survival rates after monotherapy with colistin or pogostone alone were 33.3% and 40.0%) survival at 72 h after infection of MCR-1-positve *Escherichia coli* (*E. coli)* ZJ487 (blaNDM-1-carrying), and pogostone in combination with colistin led to one or more order of magnitude decreases in the bacterial burdens in the liver, spleen and kidney compared with pogostone or colistin alone. Our results confirm that pogostone is a potential inhibitor of MCR-1 for use in combination with polymyxin for the treatment of severe infections caused by MCR-1-positive *Enterobacteriaceae*.

## 1. Introduction

The relentless rise in antibacterial resistance in extensively drug-resistant (XDR) and multidrug-resistant (MDR) Gram-negative pathogens poses a serious threat to human health. Especially in carbapenem-resistant *Enterobacteriaceae* (CRE), the treatment of drug-resistant bacterial infections is now challenging due to there being few therapeutic options [1,2,3]. Therefore, novel strategies are urgently required to address these serious clinical infections.

Colistin, a non-ribosomal cyclic lipopeptide antibiotic, is recognized as one of the last-choice antibiotics against multidrug-resistant Gram-negative bacteria [4]. At present, colistin resistance has risen along with the appearance and global spread of the mobile colistin resistance gene MCR, and additional MCR-like genes such as MCR-3 have been identified in *E. coli*, *Klebsiella pneumoniae* (*K. pneumoniae*), and/or *Salmonella typhimurium* (*S. typhimurium*) [5,6]. Notably, since its discovery in 2015, MCR-1 has spread to more than 70 countries and has engendered continuing concern in the academic world and public health communities [7]. Thus, feasible strategies are urgently needed to combat MCR-mediated acquired colistin resistance in MDR bacteria.

The development of novel antibacterial agents or antibiotic adjuvants has produced effective regimens that enhance the efficacy of antibiotics against bacterial resistance. Compared with the high costs and unpredictable nature of the development of new antimicrobial agents, the discovery of native compounds as novel and safe colistin adjuvants is a feasible prospective method [8]. As an example, pterostilbene and osthole isolated from natural plants and fruits have been demonstrated to reverse polymyxin activity and enhance the therapeutic effect of polymyxins [9,10].

Previous studies have reported that pogostone, as a primary ingredient in patchouli, possessed antioxidative [11], anti-inflammatory [12], antimicrobial [13,14] and other diverse properties. In the current study, pogostone was identified as a promising MCR-1 inhibitor that exhibited synergistic activity with colistin. Our study demonstrates that pogostone significantly reverses MCR-mediated colistin resistance both in vivo and in vitro through different mechanisms. These results suggest that pogostone, in combination with colistin, may provide an ideal approach to combatting MCR-positive bacterial infection.

## 2. Results

### 2.1. Pogostone Restored the Antimicrobial Activity of Colistin without Influencing the Growth of the Tested Bacteria

**Figure 1:**
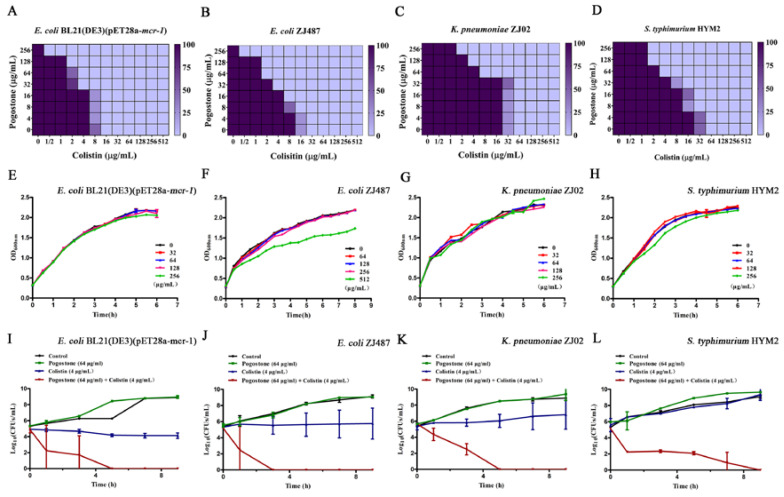
Pogostone rescues the activity of colistin. (**A**–**D**) Checkerboard MIC analysis showing the combined effect of pogostone and colistin against MCR-1-positive strains (*E. coli* BL21 (DE3) (pET28a-*mcr-1*), *E. coli* ZJ487, *K. pneumoniae* ZJ02 and *S. typhimurium* HYM2). (**E**–**H**) The growth curves *E. coli* BL21 (DE3) (pET28a-*mcr-1*), *E. coli* ZJ487, *K. pneumoniae* ZJ02 and *S. typhimurium* HYM2 under different concentrations of pogostone (0, 32–512 μg/mL). (**I**–**L**) Time-killing assays of different combinations of pogostone and colistin compared with single compound or control treatments (only medium) against *E. coli* BL21 (DE3) (pET28a-*mcr-1*), *E. coli* ZJ487, *K. pneumoniae* ZJ02, and *S. typhimurium* HYM2. Values represent the averages of three independent experiments.

*E. coli*, *K. pneumoniae*, and *S. typhimurium* are some the most common pathogenic Gram-negative bacteria in clinical practice. To determine the synergy between pogostone and colistin and to provide guidance for clinical applications, four typical MCR-1-positive strains (*E. coli* BL21 (DE3) (pET28a-*mcr-1*), *E. coli* ZJ487, *K. pneumoniae* ZJ02, and *S. typhimurium* HYM2) were examined in this study. The results of the checkerboard MIC indicated that pogostone at the concentrations of 32 µg/mL to 256 µg/mL had a significant synergistic effect with colistin in the tested MCR-1-positive bacteria (Figure 1A–D), whereas no influence of pogostone (≤256 µg/mL) on the growth was observed in the tested bacteria (Figure 1E–H). As shown in Table 1, among the tested strains, polymyxin-resistant strains (including MCR-1/3/5/8-positive strains and MCR-1/3/5/8- negative polymyxin-resistant strains) had MIC fold changes from eight to 64 at concentrations of pogostone below 64 µg/mL (FIC index from 0.17 ± 0.02 to 0.34 ± 0.05), but polymyxin-sensitive strains had MIC fold changes < 2. The pogostone MICs were ≥512 µg/mL for all tested bacteria. Therefore, pogostone could increase the antibacterial activity of colistin against most polymyxin-resistant strains.

**Figure 2:**
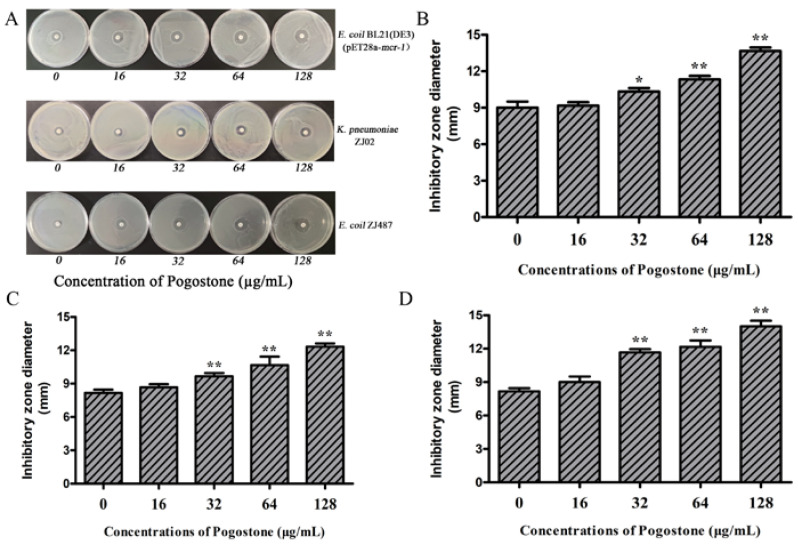
Zone of inhibition test of colistin combined with pogostone (**A**–**D**). Zones of inhibition surrounding the disks of colistin supplemented with 0, 16–128 μg/mL pogostone. Values represent the averages of three independent experiments. ** *p* < 0.01; * *p* < 0.05.

The bactericidal effect of pogostone in combination with colistin was then evaluated by time-killing assays. As expected, the tested isolates were killed completely by the combination in 9 h (Figure 1I–L). In addition, the bactericidal activity of pogostone combined with colistin was measured by the combined disk test with the measurement being the inhibition diameter on agar plates. The MCR-1-positive *E. coli* BL21 (DE3) (pET28a-*mcr-1*), *E. coli* ZJ487, and *K. pneumoniae* ZJ02 isolates had increases in the size of inhibition zones with 10 µg colistin plus pogostone (16 µg/mL, 32 µg/mL, 64 µg/mL, and 128 µg/mL) in a dose-dependent response pattern (Figure 2A–D).

**Figure 3:**
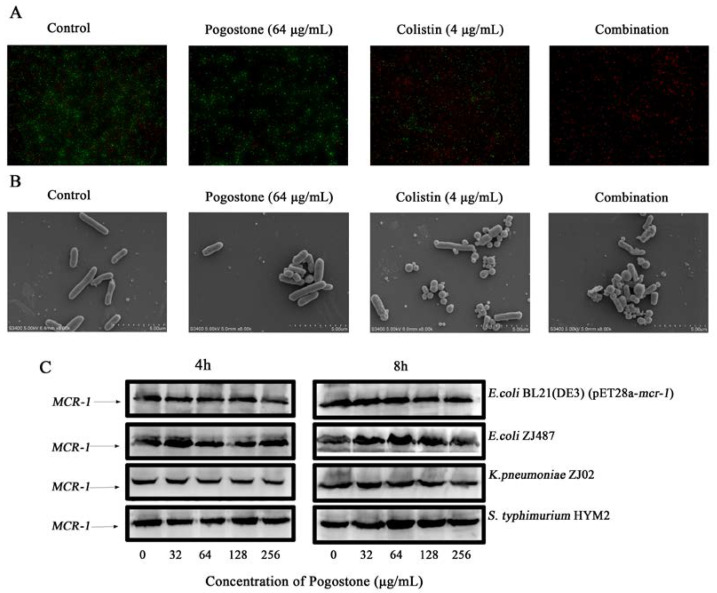
(**A**) Staining of live (green) and dead (red) bacteria. (**B**) The morphology of *E. coli* BL21 (DE3) (pET28a-*mcr-1*) observed by SEM. (**C**) MCR-1 production in *E. coli* BL21 (DE3) (pET28a-*mcr-1*), *E. coli* ZJ487, *K. pneumoniae* ZJ02 and *S. typhimurium* HYM2 treated with the indicated concentrations of pogostone (0, 32, 64, 128, or 256 μg/mL) by western blot assays.

A significant increase in the number of dead bacteria was detected by bacterial live/dead assays in the combination treatment compared to the treatment alone or the blank group (Figure 3A). The morphology of the bacteria changed significantly, with visible shrinkage of the cell walls and spherical shapes visible in dead *E. coli* BL21 (DE3) (pET28a-*mcr-1*) cells under the SEM (Figure 3B).

### 2.2. Identification of the Binding Mode of Pogostone with MCR-1/MCR-3

A western blot assay was used to confirm whether pogostone inhibited the expression of MCR-1 at different times. The MCR-1-positive strains *E. coli* BL21 (DE3) (pET28a-*mcr-1*), *E. coli* ZJ487, *K. pneumoniae* ZJ02, and *S. typhimurium* HYM2 were co-cultured with pogostone for 4 h and 8 h, and pogostone had no effect on the expression of MCR-1 (Figure 3C).

**Figure 4:**
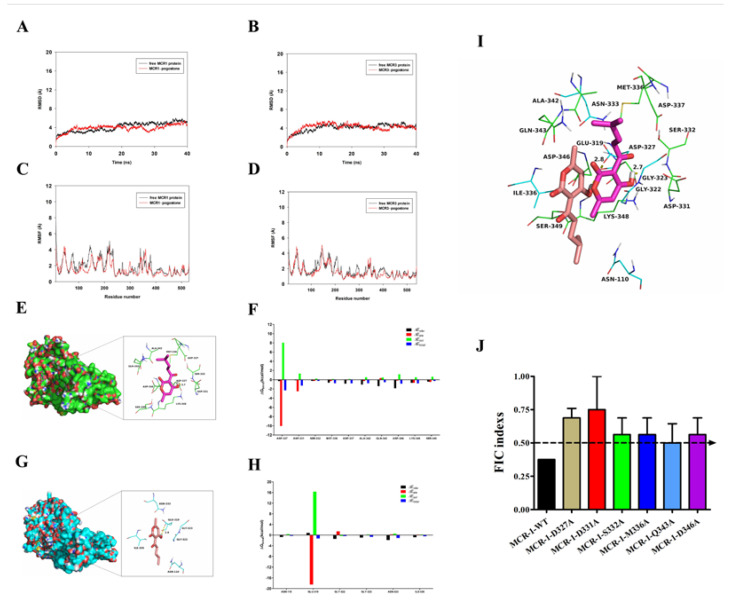
(**A**,**B**) The RMSDs of all the atoms of MCR-1/3-pogostone complexes with respect to the initial structure. (**C**,**D**) The RMSF of residues in the MCR-1/3-pogostone complex and free protein of MCR-1. (**E**,**G**) The 3D structures of MCR-1/3-pogostone complex by molecular modeling analysis. (**F**,**H**) The binding energy on a per-residue basis in the binding sites of MCR-1/3-pogostone complex. (**I**) Binding pattern diagram of MCR-1/3-pogostone complex (superposition). (**J**) The FIC indexes of pogostone with colistin of MCR-1-positive *E. coli* BL21 (DE3) (pET28a-*mcr-1*) and its MCR-1 mutants.

The root-mean-square deviation (RMSD) value of the protein backbone is an important parameter to examine whether the molecule reaches equilibrium during molecular dynamics simulations. As shown in Figure 4A,B, the MCR-1/MCR-3 system and the MCR-1-pogostone system or MCR-3-pogostone system gradually converged to equilibrium during the 40 ns simulation. To examine the fluctuations of the protein residues, we calculated the root mean square fluctuations (RMSF) of the MCR1-pogostone complex and the free MCR-1. As shown in Figure 4C,D, the RMSF was less than 2 Å when compared with the free MCR-1 or MCR-3, indicating that these residues exhibit some rigidity due to binding to pogostone.

The analysis of the binding free energy showed that residue Asp-327 was oriented to the hydroxyl group of the pogostone, forming a 2.7-Å-long hydrogen bond interaction (Figure 4E). In addition, there was a significant energy contribution from Van der Waals (LW) force(∆*E_vdw_* < −1.5 kcal/mol) for residue Asp-346 that was due to the very close proximity of residue Asp-346 to pogostone (Figure 4F). In contrast, in the MCR-3-pogostone complex, residue Glu-319 had a strong electrostatic (∆*E_ele_*) interaction with ∆*E_ele_* < −18.0 kcal/mol (Figure 4G). In addition, residue Asn-333 has a significant van der Waals energy contribution (∆*E_vdw_* < −1.5 kcal/mol) due to the very close distance between residues Asn-333 and pogostone (Figure 4H). Except for residue Asn-333, the energy contribution of most residues comes from van der Waals interactions, mainly hydrophobic interactions (including Ile-336).

By comparing the binding modes of MCR-1 and MCR-3 with pogostone (Figure 4E,G,I), we found that the hydrogen bonds formed by MCR-1 and pogostone were longer than those formed by MCR-3 and pogostone. In addition, we found that the ∆G_bind_ of pogostone was −8.5 kcal/mol and −6.1 kcal/mol for MCR-1 and MCR-3, respectively, indicating that pogostone could form stable interactions with both MCR-1 and MCR-3. In summary, the above molecular simulations yielded a rational explanation for the interactions between pogostone and MCR-1, thereby providing valuable information for further development of the MCR-1 inhibitors.

To further verify the creditability of the molecular simulations on the mechanism by which pogostone affects the biological function of MCR-1, a checkerboard MIC was applied to evaluate synergies between pogostone and colistin against the MCR-1 mutants D327A, D331A, S332A, M336A, Q343A, and D346A. As expected, the synergistic effects on mutants were reduced (FICs ≥ 0.5) compared with *E. coli* BL21 (DE3) (pET-28a-*mcr-1*) (FICs < 0.5) (Figure 4J).

### 2.3. Pogostone, Combined with Colistin, Had a Synergistic Effect Compared with Monotherapy In Vivo

**Figure 5:**
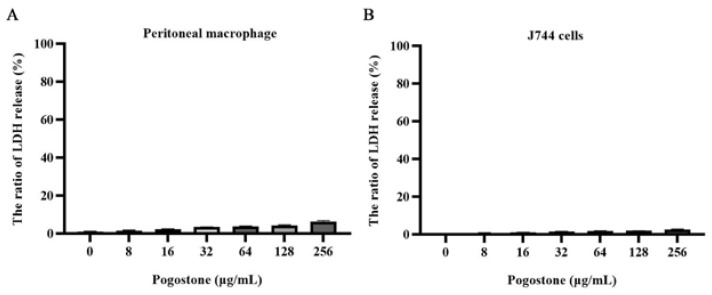
The cytotoxicity of pogostone (0–256 µg/mL) was detected by LDH release assays. LDH release assays were performed on supernatants from murine peritoneal macrophages (**A**) and J774 cells (**B**).

**Figure 6:**
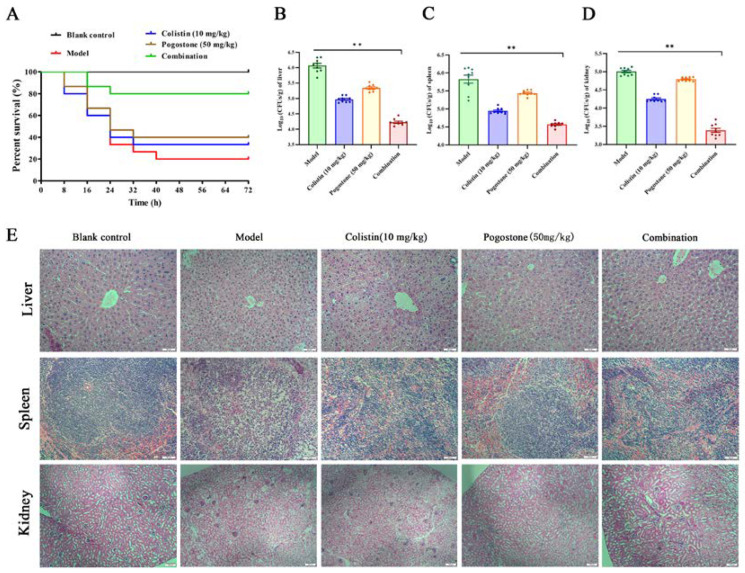
Effects of the combination therapy with colistin and pogostone in vivo. The mice were infected by intraperitoneal injection with *E. coli* ZJ478. The survival rate (**A**) and the bacterial loading of the liver, spleen, and kidney were calculated (**B**–**D**). The histopathological changes of liver, spleen, and kidney of the mice in different groups are shown in (**E**). ** *p* < 0.01.

Based on the results of minimal cytotoxicity (Figure 5A,B), we attempted to determine whether the synergistic effects could be replicated in vivo in a systemic infection model of by *E. coli* ZJ487. The result of the combination therapy was that the survival rate of mice at 72 h following intraperitoneal injection with a lethal dose of *E. coli* ZJ487 increased significantly from 20.0% to 80.0%, and the survival rates after monotherapy with colistin or pogostone alone were 33.3% and 40.0%, respectively (Figure 6A). The combination therapy showed a significant reduction of the bacterial load in the liver, spleen, and kidney compared with the monotherapy treatments or controls (Figure 6B–D), although monotherapy with colistin or pogostone showed significant decreases in CFU compared with the control group. In addition, only the combination therapy led to significant remission of liver, spleen, and kidney pathological damage, as demonstrated by histopathology (Figure 6E).

## 3. Discussion

An increasing number of human deaths from antimicrobial-resistant bacterial infections has attracted significant attention in the global community [15,16]. Thus, these infections have become a research priority of institutions for disease control and prevention. The pathogenic bacteria include methicillin-resistant *Staphylococcus aureus* (MRSA) and several multidrug-resistant (MDR) *Enterobacteriaceae* such as NDM-positive *Enterobacteriaceae* and MCR-positive *Enterobacteriaceae* [2]. Antibiotics can protect humans and animals from bacterial infections. Antimicrobial resistance limits treatment options, and this has led to increased infection-related morbidity and mortality. Of note, bacteria often possess one or more mechanisms of resistance to specific antibiotics. Polymyxins, one of the first antibiotics used in human clinical settings, have been reconsidered for use in recent years with the widespread prevalence of carbapenem-resistant *Enterobacteriaceae* (CRE). Recent studies have shown that the main mechanism by which bacteria, particularly those of the Enterobacteriaceae, develop resistance to polymyxins is the expression of the enzyme MCRs [6]. Therefore, screening for broad-spectrum MCRs inhibitors to restore the antibacterial activity of polymyxins is an effective strategy.

At present, the production of pogostone is not being considered for clinical use. Pogostone is isolated from peppermint and has antibacterial and anticancer activities [17]. It was reported that pogostone inhibits Gram-negative and Gram-positive bacteria including *Staphylococcus aureus* (*S. aureus*) and *E. coli* [18,19,20]. Intraperitoneal injection of 25 mg/kg, 50 mg/kg, and 100 mg/kg of pogostone showed antibacterial activity against *E. coli*, with 60% of mice protected by *E. coli* at 25 mg/kg, 90% at 50 mg/kg, and 100 mg/kg [13]. In our study, we also found the same protective effect after pogostone treatment alone (with 40% of mice protected from *E. coli* ZJ487 infection). However, we found that the MICs of pogostone to polymyxin-resistant *Enterobacteriaceae* were ≥512 μg/mL and were higher than the MICs of colistin (<128 μg/mL) to polymyxin-resistant *Enterobacteriaceae*. This suggests that pogostone may have different antibacterial effects in combination with polymyxin against bacterial infections.

## 4. Materials and Methods

### 4.1. Bacterial Strains and Chemicals

All of the MCR-1-positive bacterial strains and control bacteria; strains used in this study, are listed in Table 1 and were described in our previous study [10]. Pogostone was purchased from Dalian Meilun Biotechnology Co., Ltd., Dalian, China. Colistin sulfate and kanamycin sulfate were obtained from the National Institute for Food and Drug Control (Beijing, China). Dimethyl sulfoxide (DMSO; Sigma-Aldrich, St. Louis, MO, USA) was used to dissolve pogostone as a stock solution.

### 4.2. Animals and Cell Lines

BALB/c mice aged six to eight weeks and weighing 18–22 g were purchased from the Experimental Animal Center of Jilin University (Jilin, China). All the procedures involving animals and their care were conducted following the institutional guidelines and in accordance with national and international laws and policies. Mouse peritoneal macrophages and an alveolar epithelial cell line (A549, ATCC) were cultured in Dulbecco’s modified Eagle medium (DMEM) containing 10% fetal bovine serum (FBS) and penicillin–streptomycin (100 units/mL) at 37 °C with 5% CO_2_.

### 4.3. Plasmid Construction

The gene *mcr-1* was amplified from the strain *K. pneumoniae* ZJ02 using the primers listed in Table 2 and then cloned into the expression vector pET28a with the BamHI and XhoI restriction sites. The MCR-1 mutants D327A, D331A, S332A, M336A, Q343A and D346A were constructed in pET28a using a QuickChange site-directed mutagenesis kit (Stratagene, San Diego, CA, USA). All constructed strains were verified by double enzyme digestion and sequencing. The expression vectors of pET-28a-*mcr-1* and its mutants were transformed into *E. coli* BL21 (DE3) for MIC determination.

### 4.4. MIC Assays, Checkerboard MIC Assay, and FIC Index Determination

MIC assays for all the bacterial strains were determined by a method of serial two-fold dilution, essentially as described in the Clinical and Laboratory Standards Institute (CLSI) protocol [21]. For checkerboard MIC assays, 1 μL of 1 × 10^8^ CFU/mL bacterial cells was inoculated in 200 μL of LB broth containing 2-fold dilutions of pogostone (ranging from 0 μL/mL to 256 μL/mL, each column) and 2-fold dilutions of antibiotics (ranging from 0 μL/mL to 128 μL/mL, each row) and grown for 18–24 h at 37 °C in ambient air. The lowest concentrations of pogostone or antibiotics resulting in clear cultures were visually determined as the MIC values of pogostone or antibiotics. The fractional inhibitory concentration (FIC) index and the significance of the FIC index were calculated as follows:

FIC index = (MIC value of pogostone alone/MIC value of pogostone in combination) + (MIC value of antibiotic alone/MIC value of antibiotic in combination). An FIC index ≤ 0.5 was defined as synergistic; 0.5 < FIC index ≤ 4 was considered as no interaction, and an FIC index > 4 was defined as antagonistic.

### 4.5. Time Killing Assays

A time-killing study was performed on four MCR-1-positive bacterial strains. Briefly, overnight cultures of the MCR-1-positive bacterial strains (*E. coli* BL21 (DE3) (pET28a-*mcr-1*), *E. coli* ZJ487, *K. pneumoniae* ZJ02, and *S. typhimurium* HYM2) were grown to the exponential phase, and 1 μL of 1 × 10^8^ CFU/mL bacterial cells were inoculated in 96-well plates in LB medium supplemented with pogostone (64 μg/mL), colistin (4 μg/mL), colistin (4 μg/mL) combined with pogostone (64 μg/mL), or solvent control. The plates were continuously cultured at 37 °C, and samples from each group of the mixed cultures were removed and coated onto LB agar plates (containing 2 μg/mL of colistin) using a ten-fold serial dilution method to detect the number of bacteria in each group at different times (0, 1, 3, 5, 7, and 9 h). The number of colonies on the agar plates were recorded after incubation at 37 °C for 24 h.

### 4.6. Growth Curve

The bacterial strains of *E. coli* BL21(DE3)(pET28a-*mcr-1*) (Figures S1 and S2), *E. coli* ZJ487 (Figures S3 and S4), *K. pneumoniae* ZJ02 (Figures S5 and S6), and *S. typhimurium* HYM2 (Figures S7 and S8) were dissolved in LB broth and cultured at 200 rpm, 37 °C until the absorbance value reached 0.3 at OD_600_ nm. The bacterial suspension was transferred to 50 mL triangular conical flasks, and different concentrations of pogostone (0 μg/mL, 32 μg/mL, 64 μg/mL, 128 μg/mL, 256 μg/mL, and 512 μg/mL) were added. The OD_600_ nm values of samples in different groups were subsequently measured every 30 min.

### 4.7. Combined Disk Tests (CDT)

A combined disk test was used to determine the synergistic bactericidal effect of colistin and pogostone according to Zhou’s method [14]. The tested bacterial strain suspensions (OD_600 nm_ = 0.1) were dispersed onto LB agar plates, and 10 µg colistin disks (Oxoid Ltd., Basingstoke, UK) were placed in the centers. Subsequently, various concentrations of pogostone (0, 16–128 μg/mL) were added to the disks. The diameters of inhibitory zones were observed and measured after incubation at 37 °C for 24 h.

### 4.8. Bacterial Live/Dead Assays

The visual evaluation of the combined bactericidal effect of pogostone and colistin was performed by a bacterial live/dead assay. The strain of *E. coli* ZJ487 in the logarithmic growth phase was collected and divided into four groups for treatment (control, 4 μg/mL colistin, 64 μg/mL pogostone, 4 μg/mL colistin + 64 μg/mL pogostone). After incubation at 37 °C for 1 h, staining was performed according to the instructions of a BacLight Bacterial Viability Kit (Invitrogen).

### 4.9. Scanning Electron Microscope (SEM) Analysis

Overnight cultured *E. coli* ZJ487 were treated with pogostone (64 μg/mL), colistin (4 μg/mL), or a combination of pogostone (64 μg/mL) and colistin (4 μg/mL) at 37 °C for 4–5 h, washed, centrifuged, and resuspended in PBS to obtain an OD_600 nm_ of 0.5. The samples were pre-incubated with fresh PBS buffer containing polylysine (1%) and then fixed in glutaraldehyde overnight at 4 °C. The morphological changes of the bacteria were observed by SEM (Hitachi S3400, Tokyo, Japan).

### 4.10. Western Blot Analysis

Pogostone (0, 32–256 μg/mL) was added into the bacterial cultures to an absorbance of 0.1 at OD_600 nm_ and co-cultured at 37 °C for 4 h or 8 h. The samples of bacterial precipitation were added to a 5 × loading buffer and treated at 100 °C for 10 min. The prepared samples were loaded onto a 10% SDS-PAGE gel and electrophoresed at 90 V for 20 min and 140 V for 60 min. The treated samples were transferred to polyvinylidene fluoride (PVDF) membranes for 1 h and blocked with 5% skim milk powder for 30 min before being incubated overnight at 4 °C with primary mouse antibodies against MCR-1 (1:1000, prepared in 5% skim milk). The membrane was then washed and incubated with corresponding secondary antibodies (1:2000, Proteintech Group, Inc. 5500 Pearl Street, Ste 400 Rosemont, IL 60018, USA) for 1 h, developed with ECL reagent (Thermo Scientific, 81 Wyman Street, Waltham, MA 02454, USA) and visualized with a western blotting visualizer (Tanon-4200 imager).

### 4.11. Molecular Modelling

In this study, Autodock Vina 1.1.2 [22] was used for molecular docking research. The program ChemBio3D Ultra 12.0 was used to map the structure of pogostone. The data were then transformed into a three-dimensional structure using ChemBio3D Ultra 12.0, and energy optimization was performed using an MMFF94 force field. This was followed by merging non-polar hydrogen with AutodockTools 1.5.6 [23] and defining rotation keys, and finally converting to PDBQT format. The 3d structures of MCR-1 and MCR-3 proteins were modeled from homology (template: 5FGN). Both MCR1 and MCR-3 proteins were added with polar hydrogen and charge using AutodockTools 1.5.6 and finally converted to PDBQT format. The coordinates of MCR1 and MCR3 active pockets were defined as: Center_x = 49.209, center_Y = -2.988, center_z = −20.78; Size_x = 15, size_y = 15, size_z = 15. To increase the accuracy of the calculations, we set the parameter exhaustiveness to 20. We used the default values for other parameters unless otherwise specified.

Amber 14 [24,25] and AmberTools 15 software were used for molecular dynamics simulation to correct the molecular docking results. The topology file and coordinate file of compound pogostone were calculated by ACPYPE [26], a software tool based on ANTECHAMBER [27]. We applied the fields “leaprc.gaff” and “leaps.ff12SB” to small molecules and proteins, respectively. The system was then placed in a rectangular box filled with TIP3P water, the edge of which was 10.0 A from the nearest atom to the system. In order to release the tension of the system, the optimization was divided into two parts. The first part was to restrict the protein skeleton atoms and comprised 5000 optimizing steps (2500 steps for steepest descent method and 2500 steps for the conjugate gradient method). In the second part, the entire system was optimized using 5000 steps without limits (2500 steps for the steepest descent method and 2500 steps for the conjugate gradient method). Then, the system was heated from 0 K to 310 K in 1000 Ps followed by dynamic equilibrium at 500 Ps, and finally dynamics were simulated for 40 ns. The binding free energy of MCR-1 protein and small-molecule pogostone was calculated by the mm-GBSA method. The method extracted the system structure from the MD simulation trajectory of the complex at certain time intervals and calculated the average binding free energy by the following formula:

ΔG_bind_ = G_complex_ − (G_protein_ + G_ligand_),

where ΔG_bind_ is the binding free energy and G_complex_, G_protein_, and G_ligand_ are the free energies of complex, protein, and ligand, respectively.

### 4.12. Cytotoxicity Analysis

Mouse peritoneal macrophage and J744 cells were inoculated with 2 × 10^4^ cells per well in 96-well plates and incubated at 37 °C, 5% CO_2_ overnight. The test cells were washed with PBS buffer three times and cultured with different concentrations of pogostone for 6 h. Then, the supernatants were used to confirm the cytotoxicity of pogostone by lactate dehydrogenase (LDH) activity detection (Roche, Mannheim, Germany).

### 4.13. Animal Studies

The BALB/c mice were challenged intraperitoneally with a lethal dose of *E. coli* ZJ487 (2 × 10^8^ CFU) to cause a systemic infection. The infected mice were randomly divided into five groups (*n* = 15), with colistin (5 mg/kg), pogostone (50 mg/kg), a combination of pogostone (50 mg/kg) and colistin (5 mg/kg), or solvent on the same schedules. The survival status of the mice after 72 h of infection was observed and recorded.

For histopathological analysis of systemic infection, mice were inoculated with 5 × 10^7^ CFU of prepared *E. coli* ZJ487. The mice were sacrificed by anesthesia followed by cervical dislocation 48 h post infection. The liver, spleen, and kidney of the mice were placed in 10% (*v*/*v*) formalin followed by staining with hematoxylin and eosin and examination by light microscopy. The liver, spleen, and kidney of the mice in each group were harvested, homogenized, diluted, and plated to determine the respective bacterial loads 48 h post infection.

### 4.14. Statistical Analysis

All analyses were conducted using Prism v5.01 (GraphPad, La Jolla, CA, USA). The data were analyzed by using independent Student’s *t*-tests, and the results were considered statistically significantly different when the *p* value was less than 0.05.

## 5. Conclusions

In conclusion, we found a significant synergy of pogostone, in combination with colistin against polymyxin-resistant Gram-negative microorganisms, especially MCR-1-positive Enterobacteriaceae. Further studies on pogostone’s clinical treatment are warranted. Our results provide a compatibility strategy for the clinical usage of pogostone combined with colistin.

## Figures and Tables

**Table 1:** MIC values of colistin alone or combination therapy against each of the tested bacterial isolates.

Species	Source	*mcr-1*/*3*/*5*/*8* Confirmation	Antibiotics	MIC (μg/mL)		FIC Index
				Alone	Combination	
*E. coli* ZJ487	Human intra-abdominal fluid (blaNDM-1-carrying)	+	Colistin	24.00 ± 8.00	2.00 ± 0.00	0.22 ± 0.03
*K. pneumoniae* ZJ02	Remote tertiary care hospital	+	Colistin	48.00 ± 16.00	4.00 ± 0.00	0.22 ± 0.03
*E. coli* BL21(DE3) (pET28a-*mcr-1*)	Laboratory strain (carried a *mcr-1* gene that originated from *K. pneumoniae* ZJ05)	+	Colistin	12.00 ± 4.00	3.00 ± 1.00	0.31 ± 0.00
*S. typhimurium* HYM2	Animal farm	+	Colistin	48.00 ± 16.00	4.00 ± 0.00	0.22 ± 0.03
*E. coli* DZ2-12R	Chicken cloacae	+	Colistin	16.00 ± 0.00	3.50 ± 0.87	0.34 ± 0.05
*Salmonella* 15E464	I4,5,12:i:- (carried a *mcr-3* gene)	+	Colistin	56.00 ± 13.86	5.00 ± 1.73	0.22 ± 0.03
*E. coli* MG1655 (pBAD24-*mcr-5*)	Laboratory strain (carried a *mcr-5* gene)	+	Colistin	28.00 ± 6.93	1.50 ± 0.50	0.19 ± 0.04
*Salmonella* ZZW20	Animal farm (carried a *mcr-8* gene)	+	Colistin	160.0 ± 55.43	7.00 ± 1.73	0.17 ± 0.02
*K. pneumoniae* 16ZJJ9-19BC	Chicken cloacae (Polymyxin-resistant *mcr-*negative)	-	Colistin	80.00 ± 27.71	10.00 ± 3.46	0.19 ± 0.00
*E. coli* HLJ 109-11(PmrB mutation)	*pmr* B mutation (Polymyxin-resistant *mcr-*negative)	-	Colistin	448.00 ± 110.85	64.00 ± 39.19	0.27 ± 0.07
*E. coli* ATCC 25922	Laboratory strain	-	Colistin	1.50 ± 0.50	1.25 ± 0.43	1.00 ± 0.22
*K. pneumoniae* ATCC 700603	Laboratory strain	-	Colistin	1.25 ± 0.43	1.00 ± 0.00	0.94 ± 0.22
*E. coli* W3110 (pUC19-*mcr-3*)	Laboratory strain	+	Colistin	7.00 ± 1.73	1.50 ± 0.50	0.34 ± 0.05

All MICs were determined in quadruplicate. Pogostone in combination with colistin was tested at a final concentration of 64 μg/mL.

**Table 2:** The primers of *mcr-1* and its mutants used for common pcr.

Primers Name	Oligonucleotide (5′-3′)
MCR-1-D327A	F:GCGTAAGTATCTTGTGGCGTGCGAATAATTCGGACTCAAAAGGC R:GCCTTTTGAGTCCGAATTATTCGCACGCCACAAGATACTTACGC
MCR-1-D331A	F:GCGTGATAATAATTCGGCGTCAAAAGGCGTGATGG R:CCATCACGCCTTTTGACGCCGAATTATTATCACGC
MCR-1-S332A	F:CGTGATAATAATTCGGACGCGAAAGGCGTGATGGATAAG R:CTTATCCATCACGCCTTTCGCGTCCGAATTATTATCACG
MCR-1-M336A	F:GGACTCAAAAGGCGTGGCGGATAAGCTGCCAAAAG R:CTTTTGGCAGCTTATCCGCCACGCCTTTTGAGTCC
MCR-1-Q343A	F:GATAAGCTGCCAAAAGCGGCGTTTGCCGATTATAAATCC R:GGATTTATAATCGGCAAACGCCGCTTTTGGCAGCTTATC
MCR-1-D346A	F:CAAAAGCGCAATTTGCCGCGTATAAATCCGCGACCAAC R:GTTGGTCGCGGATTTATACGCGGCAAATTGCGCTTTTG
MCR-1-WT	F:CGCGGATCCATGATGCAGCATACTTCT R:CCGCTCGAGTCAGCGGATGAATGCGGT

## Data Availability

The data that support the findings of this study are available from the corresponding author upon reasonable request.

## Supplementary Materials

The following supporting information can be downloaded at: https://www.mdpi.com/article/10.3390/molecules27092819/s1, Figure S1. *E. coli* BL21(DE3) (pET28a-*mcr-1*)—(4 h); Figure S2. *E. coli* BL21(DE3) (pET28a-*mcr-1*)—(8 h); Figure S3. *E. coli* ZJ487—(4 h); Figure S4. *E. coli* ZJ487—(8 h); Figure S5. *K. pneumoniae* ZJ02—(4 h); Figure S6. *K. pneumoniae* ZJ02—(8 h); Figure S7. *S. typhimurium* HYM2—(4 h); Figure S8. *S. typhimurium* HYM2—(8 h).
