# Supplementary material for: Pogostone Enhances the Antibacterial Activity of Colistin against MCR-1-Positive Bacteria by Inhibiting the Biological Function of MCR-1
Source: Molecules. 2022 Apr 28;27(9):2819. doi: 10.3390/molecules27092819 (PMC9102576; doi:10.3390/molecules27092819)
Supplement: Supplementary file 1 [file molecules-27-02819-s001.zip › molecules-1648690-supplementary.pdf]

Supplementary Materials

Pogostone Enhances the Antibacterial Activity of Colistin against MCR-1-Positive Bacteria by Inhibiting the Biological Function of MCR-1

Shengnan Xie [1,†], Li Li [1,†], Baihe Zhan [1], Xue Shen [2,] Xuming Deng [1], Wenxi Tan [3,*] and Tianqi Fang [1,2,*]

[1] Key Laboratory of Zoonosis Research, Ministry of Education, Institute of Zoonosis, College of Veterinary Medicine, Jilin University, Changchun 130062, China
[2] Department of Food Quality and Safety, Jilin University, Changchun 130062, China
[3] Department of Obstetrics and Gynecology, The Second Hospital of Jilin University, Changchun 130041, China

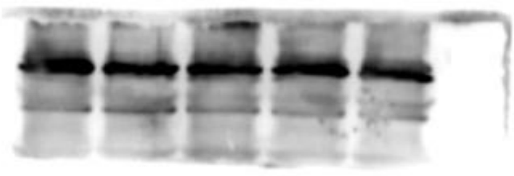

Figure S1. *E.coli* BL21(DE3) (pET28a-*mcr-1)*-(4 h).

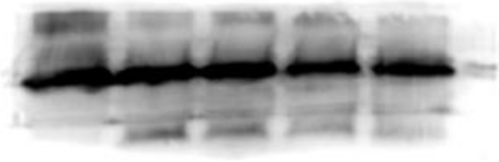

Figure S2. *E.coli* BL21(DE3) (pET28a-*mcr-1)*-(8 h).

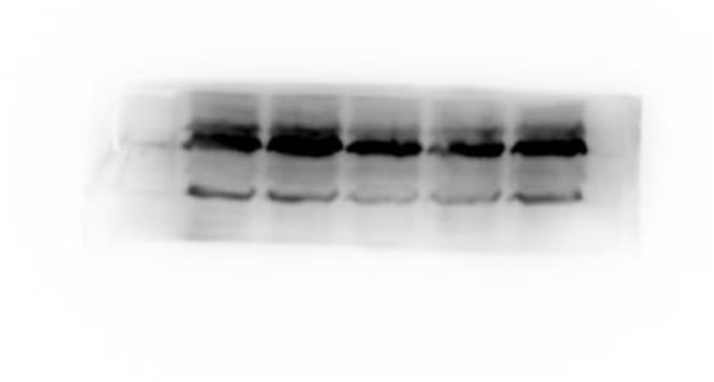

Figure S3. E.coli ZJ487-(4 h).

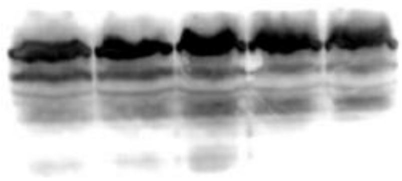

Figure S4. E.coli ZJ487-(8 h).

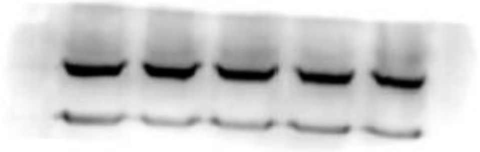

Figure S5. *K.pneumoniae* ZJ02-(4 h).

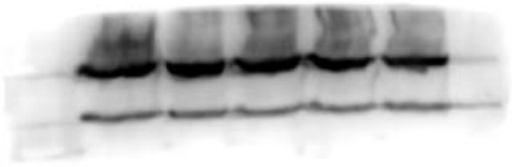

Figure S6. K.pneumoniae ZJ02-(8 h).

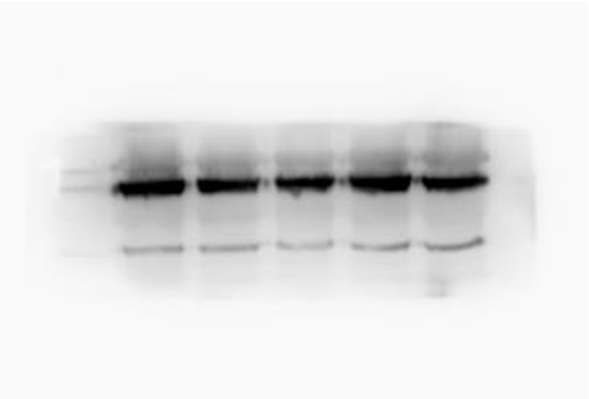

Figure S7. S. typhimurium HYM2-(4 h).

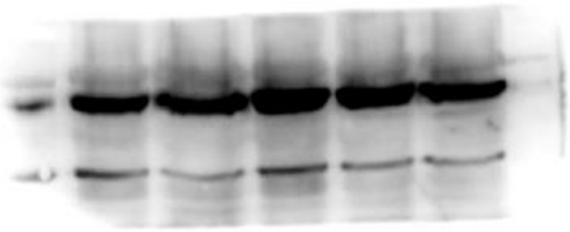

Figure S8. S. typhimurium HYM2-(8 h).
